# From Patient-Controlled Analgesia to Artificial Intelligence-Assisted Patient-Controlled Analgesia: Practices and Perspectives

**DOI:** 10.3389/fmed.2020.00145

**Published:** 2020-05-22

**Authors:** Rui Wang, Shaoshuang Wang, Na Duan, Qiang Wang

**Affiliations:** Department of Anesthesiology, Center for Brain Science, The First Affiliated Hospital of Xi'an Jiaotong University, Xi'an, China

**Keywords:** Ai-PCA, Wi-PCA, patient-controlled analgesia, artificial intelligence, postoperative pain

## Abstract

Pain relief is a major concern for patients who have undergone surgery, and it is an eternal pursuit for anesthesiologists. However, postoperative pain management is far from satisfactory, though the past decades have witnessed great progress in the development of novel analgesics and analgesic techniques. A Cochrane systematic review showed that patient-controlled analgesia (PCA) achieved better pain relief and greater patient satisfaction than traditional “on-demand” parenteral analgesia, suggesting that it might be the manner of analgesia implementation that matters for effective postoperative pain management. A wireless intelligent PCA (Wi-PCA) system that incorporated remote monitoring, an intelligent alarm, intelligent analysis and assessment of the PCA equipment, as well as automatically recording and reserving key information functions under a wireless environment was introduced in our department in 2018. The practice showed that the Wi-PCA system significantly reduced the incidence of moderate to severe postoperative pain and relevant adverse effects, shortened hospital stays, and improved patient satisfaction with postoperative pain relief. Nevertheless, for both traditional and Wi-PCA, analgesics are only administered when pain occurs, leaving behind a realm of possibilities for better postoperative pain management. With the rapid development of machinery and deep learning algorithms, artificial intelligence (AI) is changing the mode of clinical decision making. Integrating the big data collected by state-of-the-art monitoring sensors, the Internet of Things and AI algorithms, an AI-assisted PCA (Ai-PCA) may be a promising future direction for postoperative pain management.

## Introduction

Pain is a physical response to harmful stimuli and is currently perceived as a disease rather than a symptom. Acute pain after surgery is common, and severe unrelieved pain leads to not only mental stresses, such as anxiety and depression, but also physical changes in respiratory, circulatory, and immune systems, affecting the patients' life quality and recovery (1). A positive attitude toward pain management by both patients and anesthesiologists has evolved enormously over the past decades. However, the scenario of pain relief remains unchanged despite the rapid progress in the development of analgesics and the emergence of novel analgesia techniques (2). Therefore, more innovative thoughts and perspectives need to be considered to bridge the gap between the ever-growing demand for better pain control and the unsatisfactory practice of pain management.

## Unsatisfactory Progress in Postoperative Pain Relief Over the Past Decades

Ever since the publication of the acute pain management of operative or medical procedures and trauma guidelines by the Agency for Health Care Policy and Research in 1992 (3, 4), several national surveys on the epidemiology of post-surgical pain have been conducted in the United States, the United Kingdom, Canada, Germany, and France (5–11). All the surveys reported a pessimistically high incidence of moderate to severe pain in patients. The situation in China is also not optimistic. A multicenter investigation including 5,245 patients in 12 hospitals in Guangdong province showed that 56.19 and 29.73% of patients suffered from moderate/extreme pain in the first and second day after surgery (12). Another survey that included 2,293 patients from 17 hospitals in Southwest China suggested that the incidence of acute postoperative moderate to severe pain was 28.8% at rest and 45.1% in motion (13). Data from our hospital between November and December 2017 demonstrated that the overall moderate and severe pain (Numerical Rating Scale(NRS)≥4) incidence was 16.02% among 1,612 surgical patients (14).

What is more disappointing is that three national surveys conducted in the United States at ~10-years intervals showed no improvement—perhaps even worsening—in the management of postoperative pain (Table 1). In 1995, Warfield et al. surveyed 500 U.S. adults, among which 27% received surgical procedures during the past 5 years. A total of 57% of patients that underwent surgery considered postoperative pain as their major concern before surgery. More than three quarters (77%) of patients experienced different levels of pain after surgery, and the percentage of patients suffering from slight, moderate, severe, and extreme pain were 19, 49, 23, and 8%, respectively. A total of 71% of patients complained of insufficient pain relief after administration of their first pain medication (5). Apfelbaum et al. conducted the second national postoperative pain survey 8 years later in 2003. The survey included 250 randomly selected participants from the National Family Opinion-World Group, which maintains a panel of more than 550,000 U.S. households. Postoperative pain was also most concerned (59%) by the participants before surgery. The postoperative pain lasted 2 weeks after discharge for 82% of patients. Among these patients, 13% experienced slight pain, 47% experienced moderate pain, 21% experienced severe pain, and 18% experienced extreme pain. Among all the patients, ~82% received pain medication in the hospital (6). Gan et al. performed the third U.S. national survey on postoperative pain prevalence in 2014 in 300 patients. A much higher percentage (80%) of patients reported post-surgical pain as their major concern before surgery. A slightly increased percentage of postoperative pain was also observed in the survey, reaching as high as 86%. The percentage of patients reporting an intensity of pain of slight, moderate, severe and extreme was 24.5, 44.8, 22.6, and 8.2%, respectively (7).

**Table 1:** Incidence and severity of postoperative pain in three surveys conducted in the U.S.

**Pain experience**	**Warfild et al. (5) (%)**	**Apfelbaum et al. (6) (%)**	**Gan et al. (7) (%)**
Any pain	77	82	86
Slight pain	19	13	24.5
Moderate pain	49	47	44.8
Severe pain	23	21	22.6
Extreme pain	8	18	8.2

## Advantages and Flaws of Traditional Patient-Controlled Analgesia (PCA)

The innovative concept of PCA was first proposed by Sechzer et al. (15), and it was put into practice in the mid-1970s following the emergence of microprocessors. The first generation PCA employed mechanical analgesia pumps, while electronic pumps are currently playing dominant roles in clinical practice (16, 17). Several routes of PCA administration, such as intravenous, epidural, and nerve block, have also been developed, allowing for the self-administration of small doses of analgesics by patients. PCA brought pain management into a new era, shifting postoperative analgesia from “scheduled” and “requested” modes to “self-administration” modes, rendering pain control patient autonomous.

PCA has gained popularity among both patients and anesthesiologists. However, contrary to the expected high analgesia efficiency, the latest Cochrane systemic review showed that PCA only displayed marginal superiority (8%) over traditional methods of pain management (18).

One of the non-neglectable defects of the traditional PCA is that it is decentralized. PCA equipment is scattered in patient wards without direct or instant connection with medical personnel. Patients have to master the operation of the usually pre-programmed PCA equipment following a short tutorial by medical staff. There might be no problem when the equipment works properly. However, if no immediate response is made by medical staff when mechanical problems occur or when a patient's analgesia requires adjustments, the analgesia efficiency will be compromised. Moreover, the unresolved alarm sound might even trigger undesired nervous or panic emotions in patients.

## Better Performance of Wi-PCA Than Traditional PCA

The rapid development of communication technologies, especially wireless communication techniques, spawned the Internet of Things, making instant gathering and sharing information possible. The Wi-PCA that connects electronic PCA pumps and other mobile terminals with a central computer sever installed with an information control system under a wireless environment has enabled remote monitoring, intelligent alarms, intelligent analysis and assessment of the PCA equipment, and automatically recording and reserving key information (19). The Wi-PCA significantly enhanced the dynamic management of postoperative pain, and practice showed that Wi-PCA significantly reduced the incidence of moderate to severe postoperative pain as well as relevant adverse reactions, shortened the length of hospital stays, and improved patient satisfaction with postoperative pain relief compared with the traditional PCA.

Data from Cao et al. indicated that the incidence of rest (NRS ≥ 4) and motion (NRS ≥ 4) pain was significantly lower in patients using Wi-PCA in the Tumor Hospital Affiliated to Nantong University than patients using traditional PCA in 12 hospitals in Guangdong province (20), and the incidence of nausea and vomiting was significantly lower while satisfaction was higher among patients using Wi-PCA (Tables S1, S2). They also performed a historical comparison between Wi-PCA (from 2016 to 2017) and traditional PCA (in 2015) in the Tumor Hospital Affiliated to Nantong University. The data suggested that the incidence of rest pain and motion pain in the first three days after operation was significantly reduced in the Wi-PCA group, while the incidence of nausea and vomiting increased slightly (Table S3).

The Wi-PCA system was introduced to our department in the beginning of 2018. Comparison of traditional analgesic data from 1,612 patients between November and December in 2017 and Wi-PCA data from 6,191 patients between January and August in 2018 in the First Affiliated Hospital of Xi'an Jiaotong University suggested that the incidence of moderate or severe pain in surgical patients with traditional PCA was 16.02%, while the introduction of the Wi-PCA system significantly reduced the incidence to as low as 1% (Figure S1A). The patient satisfaction with pain relief was 15% higher in the Wi-PCA group (Figure S1B). Moreover, a declining trend for postoperative nausea and vomiting (PONV) was observed in contrast to Cao's observation (Figure S1C).

## Ai-PCA: a Promising Future Analgesia Direction

The Wi-PCA is still not intelligent enough, as this kind of system is not equipped with a “brain” that thinks and makes decisions independently. Indeed, to some extent, no substantial progress has been made in traditional “scheduled or requested” analgesia, traditional PCA, and Wi-PCA, as they all provide salvage analgesia instead of preventive analgesia. Preventive analgesia, a broader perioperative pain management strategy, aimed at blocking the induction of central sensitization has led to less intense pain and reduced analgesic consumption (21–23). However, in regard to postoperative PCA, no reliable parameters exist for determining the optimal time to trigger the small bolus dose of analgesic before pain occurs.

Artificial intelligence (AI) algorithms give machines the ability to reason and make decisions in a supervised or unsupervised manner. AI-powered technologies are thriving, and they are currently changing medical practice. AI has surpassed humans in several medical areas, such as disease diagnosis based on medical or pathological images and disease activity monitoring for atrial fibrillation and epilepsy relapse (24–29). Pioneering work of AI applications in anesthesiology has been carried out in several aspects, including anesthesia depth monitoring, control of anesthesia, risk prediction, and logistics management (30). In pain management, AI has been used to select patients who may benefit from preoperative pain consultation (31).

**Figure 1:**
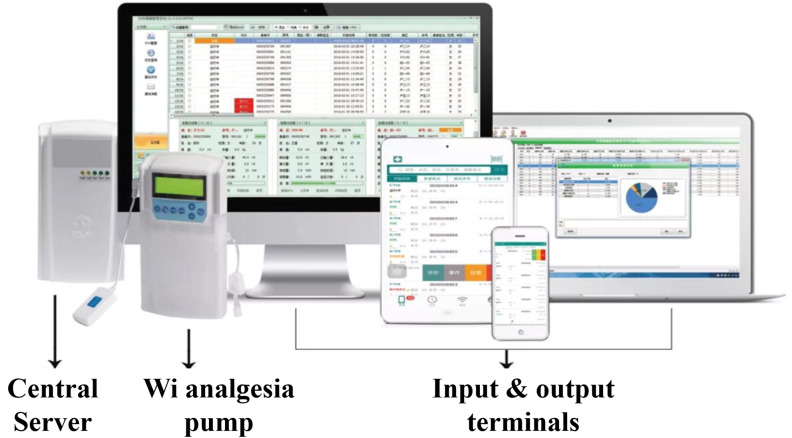
The composition and features of Wi-PCA.

Like the biological neural circuit, an integrated AI application requires both input and output terminals apart from the central model built with specific AI algorithms. Indeed, the Wi-PCA provides the hardware structure of Ai-PCA, that is the computer server and the terminals that send signals to and receive signals from the server (Figure 1). Ai-PCA requires more specific detectors that gather and send more specific signals to the server and equips the server with an AI model.

Choosing the right parameter as the input signal is key in Ai-PCA development. The preventive analgesia concept is based on the “injury discharge” phenomenon, a basic science discovery that peripheral nerve injury-triggered afferent barrage consists of high-frequency bursts which are different from normal neural responses by natural stimuli (32). The “injury-discharge” might be a good candidate, but the feasibility depends on its accessibility by wearable detectors. Hence, cooperation between basic and applied scientists are necessary for successful Ai-PCA development.

Another issue in AI applications in pain management is ethical and safety concerns, although the use of AI applications in postoperative pain management is an irresistible trend. Future disputes might be focused on whether the best course of action is the use of Ai-PCA or AI-controlled analgesia.

## Conclusions

The disappointing fact that almost no real progress has been made in the past two decades in postoperative management requires innovation in the development of analgesia strategy. AI is a promising approach to shift salvage analgesia to a preventive era.

## Data Availability Statement

All datasets generated for this study are included in the article/Supplementary Material.

## Author Contributions

QW, RW, SW, and ND contributed conception and design of the study. SW organized the database. RW and ND performed the statistical analysis. RW wrote the manuscript. All authors contributed to manuscript revision, read, and approved the submitted version.

## Conflict of Interest

The authors declare that the research was conducted in the absence of any commercial or financial relationships that could be construed as a potential conflict of interest.
