# Supplementary material for: From Patient-Controlled Analgesia to Artificial Intelligence-Assisted Patient-Controlled Analgesia: Practices and Perspectives
Source: Front Med (Lausanne). 2020 May 22;7:145. doi: 10.3389/fmed.2020.00145 (PMC7326064; doi:10.3389/fmed.2020.00145)
Supplement: Supplementary file 1 [file Table_1.docx]

**Table S1. Incidence of postoperative pain and side-effects first after operation for traditional PCA (Guangdong) and Wi-PCA (Nantong)**

| Group | NRS≥4 | | NRS≥5 | | Oversedation | Nausea & vomiting | Patient satisfaction |
| --- | --- | --- | --- | --- | --- | --- | --- |
|  | Rest pain | Motion pain | Rest pain | Motion pain |  |  |  |
| Guangdong  Hospitals  (n=1235) | 12.06 | 39.03 | 4.94 | 20.08 | 5.10 | 20.65 | 97.73 |
| Tumor Hospital of Nantong University  (n=6601) | 0.38^*^ | 13.32^*^ | 0.15^*^ | 3.33^*^ | 1.18^*^ | 5.85^*^ | 99.92^*^ |

^*^ Compare 2016, 2017 with 2015，^*^ P <0.05
