# Supplementary material for: From Patient-Controlled Analgesia to Artificial Intelligence-Assisted Patient-Controlled Analgesia: Practices and Perspectives
Source: Front Med (Lausanne). 2020 May 22;7:145. doi: 10.3389/fmed.2020.00145 (PMC7326064; doi:10.3389/fmed.2020.00145)
Supplement: Supplementary file 2 [file Table_2.docx]

**Table S2. Incidence of postoperative pain and side-effects second after operation for traditional PCA (Guangdong) and Wi-PCA (Nantong)**

| Group | NRS≥4 | | NRS≥5 | | Oversedation | Nausea & vomiting | Patient satisfaction |
| --- | --- | --- | --- | --- | --- | --- | --- |
|  | Rest pain | Motion pain | Rest pain | Motion pain |  |  |  |
| Guangdong  Hospitals  (n=1235) | 9.80 | 17.33 | 5.02 | 8.10 | 1.13 | 6.40 | 90.33 |
| Tumor Hospital of Nantong University  (n=6601) | 0.14^*^ | 10.68^*^ | 0.09^*^ | 2.32^*^ | 1.88 | 4.79^*^ | 99.98^*^ |
