# Supplementary material for: From Patient-Controlled Analgesia to Artificial Intelligence-Assisted Patient-Controlled Analgesia: Practices and Perspectives
Source: Front Med (Lausanne). 2020 May 22;7:145. doi: 10.3389/fmed.2020.00145 (PMC7326064; doi:10.3389/fmed.2020.00145)
Supplement: Supplementary file 3 [file Table_3.docx]

**Table S3. Historical comparation of Incidence of postoperative pain and side-effects for traditional PCA and Wi-PCA**

| Group | NRS≥4 | | NRS≥5 | | Oversedation | Nausea & vomiting |
| --- | --- | --- | --- | --- | --- | --- |
|  | Rest pain | Motion pain | Rest pain | Motion pain |  |  |
| PCA (2015) | 1.22 | 32.2 | 0.63 | 10.67 | 4.59 | 9.19 |
| PCA (2016) | 0.51^*^ | 22.91^*^ | 0.23^*^ | 4.23^*^ | 2.93^*^ | 10.55 |
| PCA (2017) | 0.36^*^ | 21.23^*^ | 0.22^*^ | 3.86^*^ | 5.16^*^ | 13.42^*#^ |

^*^Compare 2016, 2017 with 2015，^*^ P <0.05； ^#^Compare 2017 and 2016，^#^ P <0.05
