# Supplementary material for: From Patient-Controlled Analgesia to Artificial Intelligence-Assisted Patient-Controlled Analgesia: Practices and Perspectives
Source: Front Med (Lausanne). 2020 May 22;7:145. doi: 10.3389/fmed.2020.00145 (PMC7326064; doi:10.3389/fmed.2020.00145)
Supplement: Supplementary file 4 [file Image_1.pdf]

Figure S1

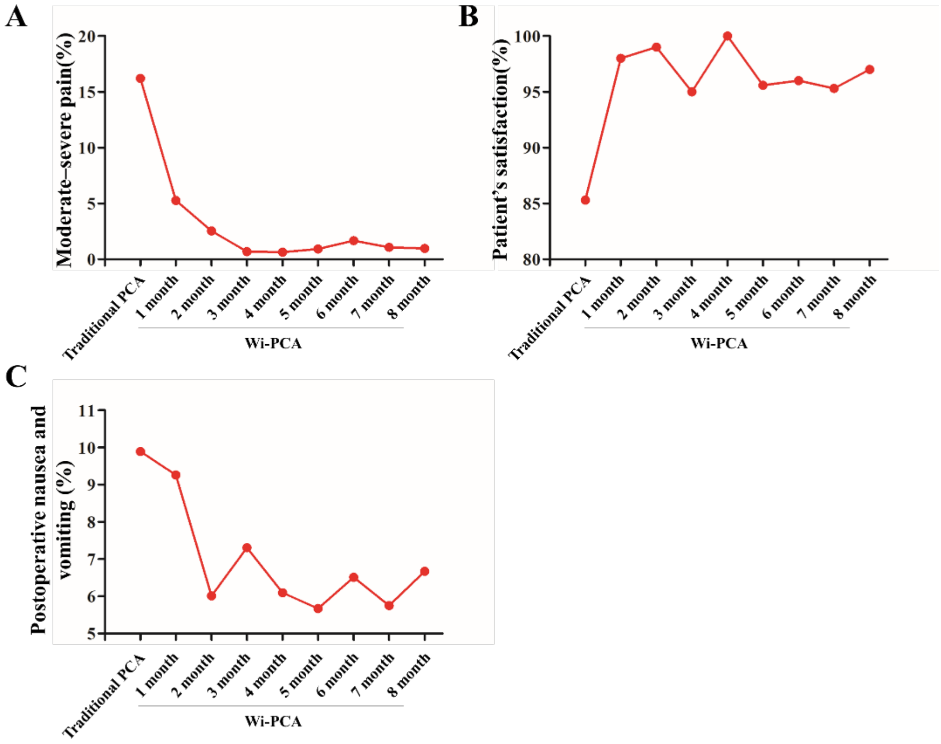

Figure S1. Effect of Wi-PCA on pain, patient satisfaction and PONV after surgery. A. Wi-PCA significantly reduce the incidence of moderate or severe pain after surgery. **B**. Wi-PCA significantly improve patient satisfaction for pain relief after surgery. **C**. Wi-PCA decrease incidence of postoperative nausea and vomiting (PONV) after surgery
